# 

**Published:** 1898-07

**Authors:** 


### Communicated.

### SEVENTH ANNUAL COMMENCEMENT, DENTAL DEPARTMENT, UNIVERSITY OF BUFFALO.

BUFFALO, N. Y., May 9th, 1898.

THE AMERICAN JOURNAL OF DENTAL SCIENCE :

The seventh annual commencement exercises of the Dental Department of the University of Buffalo was held in connection with those of the Departments of Medicine and Pharmacy at Music Hall in the city of Buffalo.

Upon the recommendation of the Faculty and by vote of the Council, the Chancellor conferred upon the following students, who had complied with all the requirements, the Degree of Doctor of Dental Surgery :

George Avereil, New York; Edward Barber, Ontario; Edgar Bartlett, Ontario; Charles Borland, New York; William Bostwick, New York; Byron Brown, New York; Jacob Brown, New York, Charles Buckland, New York; Harry Burghardt, New York; Egerton Cays, Ontario, Claude Christopher, New Yoik; John Cole, Ontario; Arthur Consanl, Ontario; Herschel Cull; New York; Harry Day, New York; George Decker, New York; William Dills, New York, Kernon Donahue, New York; John Dudley, Ontario; Moss Eshleman, New York; Raymond Evans, New York; Frank Frank, New York; Katharine Graf, New York; Allen Green, New York; John Hanna, New York; Albert Heist, Ontario; Harry Howell, Ontario, George Jones, New York; Albert Jung, New York, Otto Kalbfleisch, Ontario; Henry Kitching, New York; Charles Laborn, New York; Christian Landel, New York; Clarence Lauderdale, New York; Clinton McCallum, Ontario; Arthur Mix, New York; Herbert Morris, Ontario; Robert Murray, Ontario; Judson North, New York; George Northup, New York; William Paul, New York; John Quigg, New York; Harry Randall, Pennsylvania; Grace Rankin, Ohio; Howard Redfield, New York; Earnest Rice, Ontario; Samuel Salisbury, New York; Edward Schlottman, New York, William Sheahan,

Ontario; William Slacer, New York; Miles Smith, New York; Josephine Spellman, New York; Louis Squires, Ph. B., New York; Ella Staley, New York; George Stevenson, Ontario; Duncan Stewart, Ontario; Eli Sweet, New York; Charles Tefft, New York; John Tooke, New York; George Van Marter, New York; Francis Wallace, Ontario; Louis Wettlaufer, Ontario; Jay Wilson, New York; Fred. Woodmansee, Pennsylvania; William Worrell, New York.

### To Readers and Correspondents.

Communications intended for publication, and exchange journals and papers, should be addressed to the Editor of The American Journal of Dental Science, No. 845 N. Eutaw St. Baltimore, Md. All letters relating to the business of the Journal, or containing cash remittances, to be directed to Snowden & Cowman Mfg. Co., 9 W. Fayette Street, Baltimore, Md. Articles for publication should be received by the Editor at least two weeks previous to the first of the month on which the number in which it is designed for them to appear is issued.

☞ The American Journal of Dental Science will contain fifty pages of reading matter in each number, making a volume of about Six Hundred pages,

EXCLUSIVE OF ADVERTISEMENTS.

